# Lethal and Sublethal Responses of *Hydropsyche pellucidula* (Insecta, Trichoptera) to Commercial Polypropylene Microplastics after Different Preconditioning Treatments

**DOI:** 10.3390/toxics9100256

**Published:** 2021-10-09

**Authors:** Manuela Piccardo, Marco Bertoli, Paolo Pastorino, Damià Barceló, Francesca Provenza, Davide Lesa, Serena Anselmi, Antonia Concetta Elia, Marino Prearo, Elisabetta Pizzul, Monia Renzi

**Affiliations:** 1 Department of Life Sciences, University of Trieste, Via L. Giorgieri 10, 34127 Trieste, Italy; manuela.piccardo@phd.units.it (M.P.); marco.ber3@gmail.com (M.B.); francesca.provenza@phd.units.it (F.P.); davidelesa@libero.it (D.L.); pizzul@units.it (E.P.); mrenzi@units.it (M.R.); 2 The Veterinary Medical Research Institute for Piemonte, Liguria and Valle d’Aosta, Via Bologna 148, 10154 Torino, Italy; marino.prearo@izsto.it; 3 Catalan Institute for Water Research (ICRA-CERCA), Carrer Emili Grahit 101, 17003 Girona, Spain; dbcqam@cid.csic.es; 4 Institute of Environmental Assessment and Water Research (IDAEA-CSIC), Jordi Girona 18-26, 08034 Barcelona, Spain; 5 Bioscience Research Center, Via Aurelia Vecchia 32, 58015 Orbetello, Italy; serena.anselmi@bsrc.it; 6 Department of Chemistry, Biology and Biotechnology, University of Perugia, Via Elce di Sotto 8, 06123 Perugia, Italy; antonia.elia@unipg.it

**Keywords:** conditioning, ecotoxicological impact, macroinvertebrates, microplastics, riverine water, surfactant

## Abstract

Microplastics (MPs) pose biological and chemical hazards in aquatic and terrestrial food webs across the globe. Research on microplastic contamination has long focused on marine ecosystems, whereas the toxicological impact on freshwater organisms is still little explored. In this study, the lethal and sublethal response of the freshwater macroinvertebrate *Hydropsyche pellucidula* exposed to polypropylene MPs after different pre-conditioning treatments was assessed. Field samples were collected in a riverine system (Vipacco river; northeast Italy) to assess the characteristics of the MPs in the aquatic environment Both water and sediment were contaminated by MPs (3.73 ± 2.11 items m^−3^ per min and 3.33 ± 4.16 items dm^−3^, respectively). The chemical MPs composition included polystyrene, polyethylene terephthalate, polyurethane, polyamide, polypropylene, and polyethylene. Polypropylene (PP), although not the most abundant polymer recorded in the study area, was preferred over the other types according to its abundance in freshwater and *H. pellucidula* feeding behavior. A housing test was performed to recreate the natural conditions of larvae sampled for a reliable response to the ecotoxicological tests. The microplastics underwent either preconditioning with Vipacco River water (PP-river) and surfactant Triton X-100 (PP-sf) or no pre-treatment (PP). Submersion of microplastics in 10 µg L^−1^ of surfactant solution for 24 h was sufficient to induce consistent spectral changes and modify the chemical profile of the plastic surface. Mortality rate differed according to treatment: PP and PP-river > positive control > PP-sf > negative control. Integrated biomarker response (IBRv2) and analysis of oxidative stress biomarker levels showed a greater response of superoxide dismutase and lipid peroxidation (malondialdehyde) in larvae treated with PP conditioned in surfactant. Our findings enhance knowledge on the toxicity of PP and conditioning phases on *H. pellucidula* larvae.

## 1. Introduction

Microplastics are a new type of pollutants composed of tiny plastic fragments less than 5 mm in any dimension and a generally accepted lower limit of 1 µm [1,2]. Ubiquitous in the environment, microplastics from human activity are found in terrestrial [3] and in freshwater ecosystems: lakes [4,5,6]; rivers [7,8]; estuaries [9]; groundwater [10] and wastewater [11]. Freshwater and terrestrial environments are recognized entry and transport pathways of plastics to oceans, however, plastic debris in freshwater systems remains understudied: 87% of plastic pollution studies concern marine environments and 13% freshwater systems [12]. Our knowledge of microplastics contamination of terrestrial and freshwater ecosystems is scarce, fragmented, or even absent for some countries [12]. Where data are more consistent, the level of microplastics concentration varies, spanning ten orders of magnitude (1 × 10^−2^ to 10^8^ particles/m^3^) [13]. There is an urgent need to improve current knowledge, acquire environmental data on microplastics contamination in freshwater systems, and explore its toxicological impact.

A potential risk posed by microplastics in the environment is their bioavailability to aquatic organisms. Research on marine species has reported ingestion of microplastic particles by a wide range of species at various trophic levels and with different feeding strategies [14]. Given the similarity of some phyla common to both freshwater and marine ecosystems, similar outcomes of ingestion are almost inevitable. Potential hazards to freshwater organisms after the ingestion of microparticles include reduced food assimilation efficiency in *Gammarus fossarum* (Amphipoda) [15]; reduced growth and reproduction in *Hyalella azteca* (Amphipoda) [16]; immobilization in *Daphnia magna* (Crustacea) [17]; inflammation, altered gut microbiome and tissue metabolic profile, genotoxicity, and immune toxicity in zebrafish [18,19,20].

Likewise, toxicological interaction of microplastics with co-contaminants (e.g., heavy metals, polycyclic aromatic hydrocarbons, pharmaceuticals, pesticides) [21,22,23] may promote alteration of important protective metabolic pathways in marine and freshwater organisms. Oxidative stress promoted by contaminants may be counterbalanced by cellular antioxidant scavengers (superoxide dismutase (SOD), catalase (CAT), glutathione peroxidase (GPx)) and associated (glutathione S-transferases (GST), lipid peroxidation) enzymes/metabolites. Triads of antioxidant enzymes are key components within the primary defense system against reactive oxygen species (ROS), catalyzing the breakdown of ROS-generating O_2_^−^ (SOD) and hydrogen peroxide (CAT and GPx). The phase II enzyme glutathione-s-transferase (GST) catalyzes the conjugation of GSH to the electrophilic centers of a wide range of substrates by sulfhydryl groups, preventing their interaction with biomolecules.

Moreover, microplastics may also pose a chemical hazard. Plastic debris is often composed of a complex mixture of chemicals: ingredients of the plastic material (monomers and additives) and by-products of manufacturing and chemical contaminants in water that accumulate on plastic when it becomes litter (persistent organic pollutants [POP] and metals) [1,24]. The latter may be especially hazardous for inland freshwater systems, where chemical concentrations are estimated to be higher than in marine systems due to the continuity of use of these chemicals [25]. Several chemicals in plastics have been identified as either toxic or endocrine disruptors, including bisphenol-A, phthalates, polybrominated diphenyl ethers (PBDEs), and metals [26]. The chemicals are weakly bound or not bound to the polymer molecule and so may leach out of the plastic over [14]. This phenomenon makes the interactions between microplastics and organic pollutants particularly pertinent in inland freshwater systems, especially in those near industrialized and populated areas [14].

Chemicals-plastics sorption depends on multiple factors: polymer type, particle size, and physicochemical characteristics (i.e., water pH, salinity, temperature, presence of organic matter) of the site where the plastics become litter [24]. Furthermore, surfactants may act as agents that sequester some of the chemical species associated with microplastics, for example by increasing the leaching concentration of POPs [27]. Surfactants are one of the most widely used families of organic compounds added to the formulation of cosmetics, personal care products, textiles, dyes, food, agrochemicals and oils [28]. A fundamental property of surfactants is their ability to form micelles in solution via hydrophobic and hydrophilic groups. This feature gives surfactants their detergency and solubilization properties and affords them a major role in the chemical risk of microplastics. In brief, before conducting toxicological evaluation, the conditioning phase of plastics in solutions needs to be considered.

Macroinvertebrates represent reliable indicators of freshwater quality given their tolerance toward a variety of environmental emergent contaminants [29] including microplastics [30,31]. The caddisfly *Hydropsyche* of the order Trichoptera (Insecta), is a univoltine genus, net-spinning caddisfly collector-gatherer (sometimes reported as a filterer) in the functional feeding guild [32]. It is a clinger organism that builds a net to catch its food, which consists of algae, small invertebrates, and detritus. It is a critical component of biomonitoring programs throughout its geographical range because of its high abundance and ample range of pollution tolerance [33]. *Hydropsyche* larvae are widespread inhabitants of temperate freshwater streams where they are used as sensitive biomonitors in the field [34] and in laboratory studies on chemical stress from metal contamination due to acid mine drainage [35], fenvalerate (a synthetic pyrethroid insecticide) [36], industrial wastes, and agricultural runoff [37].

Herein, a laboratory investigation was undertaken evaluating the toxicity of microplastics on *H. pellucidula* larvae collected from a riverine system (Vipacco river) following preconditioning in riverine water and in a surfactant solution of Triton X-100 (representative of surfactant-like substances). The aims of the present study were: (a) determine the environmental characteristics (shape, size, color and chemical type) of microplastics in water and sediment samples from Vipacco river; (b) determine whether conditioning modified the chemical profile (spectral changes) and the size of the microplastics particles and (c) measure the lethal and the sublethal response in terms of oxidative stress of the freshwater macroinvertebrate *H. pellucidula* exposed for 10 days to conditioned and unconditioned microplastics.

## 2. Materials and Methods

### 2.1. Determination of Environmental Microplastics Levels

#### 2.1.1. Vipacco River

The Vipacco is the main left tributary of the Isonzo River [38]. It originates from karstic springs at Mount San Lorenzo in Slovenia (1019 m a.s.l.), where its course runs for 45 km in Slovenia, then crosses the border with Italy and continues for 4.5 km within the municipality of Savogna d’Isonzo before emptying into the Isonzo. The Italian stretch of the Vipacco is an interesting sampling site: the slightly modified watercourse flows across a high plain that is largely rural, with some agricultural/small industrial activity and wastewater discharge. The Vipacco is included in the monitoring framework of the Regional Agency for Environmental Protection of Friuli-Venezia Giulia (ARPA FVG) and is sensitive to anthropic pressures and impacts from the upstream area in Slovenia (http://www.arpaweb.fvg.it) (accessed on 29 September 2021).

#### 2.1.2. Sediment Sampling

In October 2020 sediment samples (*n* = 3) were collected with a manual corer (250 cm^2^ sampling surface) from the riverbed near the banks at a sampling site (45°53′17.670″ N; 13°34′47.800″ E) that can be submerged during extreme low-level water periods. The samples were placed in closed glass jars (1 L) to protect them from external particle contamination, frozen at −20 °C for storage, and thawed prior to extraction and determination of the chemical composition of the plastic and the microplastics.

#### 2.1.3. Water Sampling

Water samples (*n* = 3) were collected with an Apstein plankton net (opening 400 × 1000 mm; mesh size 50 μm) at the same site and time as the sediment samples by placing the net directly below the water surface. The net was kept in place for 30 min per sample. Water volume was calculated using a manual flowmeter (fixed on the plankton net opening) [39]. The water samples were transferred into glass jars. The net was cleaned between each replicate with ultrapure water to prevent contamination of the subsequent sample. The water samples were used to determine plastic and the microplastic chemical type and physicochemical features of the water.

#### 2.1.4. Sample Extraction and Sorting

The samples were treated and analyzed according to published methods [13,40]. Positive and negative controls were performed during extraction and quantification to avoid false positives and false negatives (*n* = 5). Laboratory analysis was performed under a HEPA-II hood. The optimal digestion method for the matrix was selected [40]. The water samples were filtered by vacuum on 10-μm pore paper disk filters (Whatman^®^, Sigma-Aldrich, St. Louis, MO, USA). The filters were placed on a glass Petri disk to avoid pollution during oven drying (35 °C); after water evaporation, the filters were analyzed by stereomicroscopy to collect plastic and microplastic particles for chemical determination. Sediment samples were extracted three times using a prefiltered saturated solution of NaCl by mechanical agitation (20 min, 100 rpm) and the supernatant was filtrated on 10-μm pore paper disks.

#### 2.1.5. Chemical Analysis by Fourier-Transform Infrared Spectroscopy

The plastic and the microplastics were analyzed by microscopy coupled with Fourier transform infrared spectroscopy (μFT-IR Nicolet iN 10MX^®^, Thermo Fischer Scientific, Waltham, MA, USA). The samples were analyzed by reflection using a gold plate as reflecting substrate or by transmission using BaF2 windows (Thermo Fisher Scientific). The μFT-IR system was equipped with a liquid nitrogen-cooled MCT-A detector (spectral range 7800–650 cm^−1^) operated via an OMNICTM Picta (Thermo Fisher Scientific) user interface. Chemical spectra were acquired and compared to a library of known materials to determine spectral differences. The mean spectrum was calculated for each particle by plotting 10 single spectra acquisitions together to obtain a robust spectral representation of a single particle. The limit of detection (LOD) for chemical analysis of particles was 10 μm; smaller size particles were not identified for sediments. The limit of quantification (LOQ) of the water samples was determined by the 50-μm mesh of the sampling plankton net used for the field activities.

### 2.2. In Vivo Experiment and Exposure

#### 2.2.1. Hydropsyche Pellucidula Larvae Sampling

In November 2020, macroinvertebrates were sampled from the Vipacco River using a Surber net (mesh 250 μm) following a standardized method for macroinvertebrate biomonitoring in wadable rivers [41]. In order to obtain a sufficient number of specimens, the larvae were collected with steel entomological tweezers. The specimens were sorted in the field; 225 *H. pellucidula* larvae (homogeneous in size; 1.7 ± 0.56 cm) were collected, transported to the laboratory in glass jars (1 L) filled with riverine water, and stored in glass tanks with 3 L riverine water (Vipacco) for 10 days acclimatization before the experiment.

#### 2.2.2. Housing Test and Experimental Design

A housing test was run to recreate the best natural conditions and not diminish the reliability of the toxicological test responses. The test entailed housing the larvae in a glass aquarium (27 × 27 × 16 cm) containing 3 L of Vipacco River water; the aquarium was equipped with an aeration system and enriched with the addition of leaves of *Salix* sp., *Acer* sp., and *Alnus glutinosa* (one leaf per species) collected in the field. The addition of the leaves created a mesocosm familiar to the larvae, reduced stress, and provided a source of food and/or supporting substrate.

#### 2.2.3. Reference Microplastics and Surfactant

**Figure 1:**
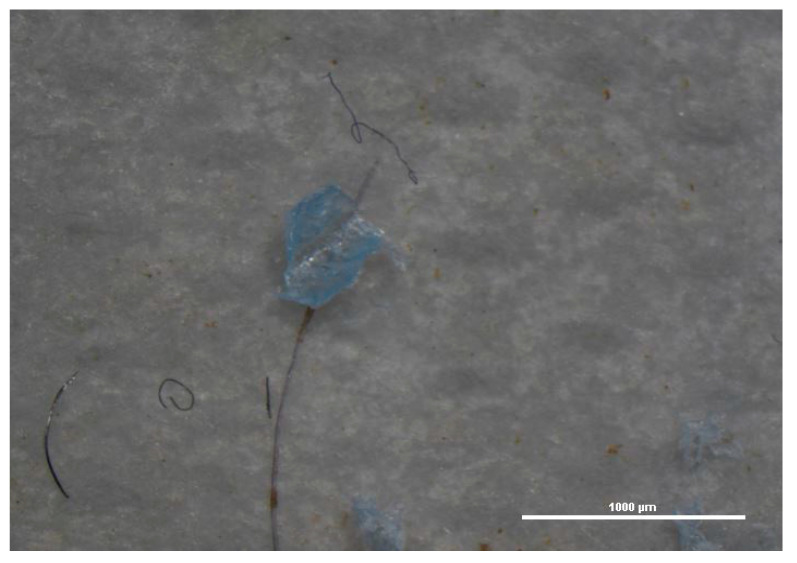
Irregular blue fragment of polypropylene used as reference material.

Polypropylene (PP), although not the most abundant polymer in the study area (see Section 3.1), was recorded in both sediment and water samples from the Vipacco. On this path, PP was selected as reference material for the exposure test according to both its abundance in freshwater [42] and *Hydropsyche* behavior (being both a collector-gatherer and filterer organism) [32]. Indeed, according to the literature [42], PP is one of the main MP contaminants in freshwater and sediments. There are several particle characteristics of polypropylene that cause it to repel water and float. However, PP was conditioned both in riverine water (Vipacco river) and surfactant. The presence of both algae/biofilm and surfactant material would cause the particle to be at least partially soluble in the surrounding water and would change the surface energy enough to allow water to penetrate causing the sinking of the particle [43,44]. Irregular shaped PP fragments (Figure 1) (mean size 377.74 ± 168.09 µm; mean ± standard deviation; min 179.74 µm; max 497.80 µm) were produced in the laboratory by shredding commercial stoppers, sieved to the required size, washed with deionized water, oven-dried at 30 °C, and stored in falcon tubes until use.

Triton X-100 (micellar average MW 80,000; average MW 625), a common non-ionic surfactant and emulsifier was purchased from Sigma Aldrich and used as a reference surfactant for preconditioning a test group of microplastics and for preparing the positive control. The concentration (10 µg L^−1^) was selected because far below the LC_50_ determined in a preliminary test for *H. pellucidula* larvae (LC_50_ 2.70 mg L^−1^; 96 h).

#### 2.2.4. Microplastics Conditioning and Post-Treatment µFT-IR Characterization

The microplastics for the toxicological test underwent three different pre-treatments before exposure to the larvae: one group of microplastics was conditioned by immersion in Vipacco riverine water (directly on-site inside a woven nylon mesh (<10 µm)) for 15 days (PP-river), one group was conditioned in a solution (prepared with ultrapure water) of 10 µg L^−1^ Triton X-100 for 24 h (PP-sf), and a third group received no pre-treatment (PP). The PP-river and the PP-sf group were filtered on paper disks and then characterized by μFT-IR analysis to detect differences in microparticles, as previously reported. The conditioned and the unconditioned microplastics were analyzed to collect and compare FT-IR spectra.

#### 2.2.5. Experimental Design

Specimens of *H. pellucidula* (*n* = 225) after acclimatization were then randomly distributed to set up five experimental groups: a negative control (Vipacco water; Control−); positive control (Triton X-100; Control+); untreated plastics (PP); plastics preconditioned in riverine water (PP-river), and plastics preconditioned in Triton X-100 solution (PP-sf); 125 µg L^−1^ of microplastics were added once at the beginning of the 10-day experiment. The exposure concentration was based on environmental concentrations of microplastics reported for the Vipacco river [45].

Each experimental set-up consisted of three aquaria (replicates) with 15 larvae per aquarium, for a total of 225 larvae in the entire experiment.

During exposure, the main physicochemical parameters of water (temperature, pH, conductivity, and oxygen) were monitored daily using multiparametric probes (HI 9125 pH/ORP meter, HI 9147 dissolved oxygen meter, Hanna Instruments Inc., Woonsocket, RI, USA). The photoperiod was kept constant (12 h light/12 h dark).

The aquaria were checked every 6 h and mortality was recorded daily. At the end of the experiment (10 days), live larvae were euthanized and stored at −80 °C until biochemical analysis and quantification of microplastics body burden.

#### 2.2.6. Microplastics Extraction and Quantification in Biota

One pool of larvae per experimental set (*n* = 3) was added to H_2_O_2_/H_2_O (1:1 *v*/*v*, 15% final H_2_O_2_ concentration) and digested by an ultrasonic bath (40 KHz, 20 min). The digested samples were filtered on Anodisc^®^ filters (Sigma-Aldrich) and analyzed by μFT-IR (transmission mode) to determine ingestion of PP microparticles.

#### 2.2.7. Biochemical Analysis and IBRv2 Index

Each aquarium (replicates; *n* = 3) was analyzed separately. The larvae from each replicate were pooled together into two groups. The negative controls were evaluated for natural response. Biochemical analysis was performed on the whole body of the live larvae using protein fraction S9 [46]. A buffer phosphate 50 mM + EDTA (2 mM) was added at a set ratio in a test tube containing the larvae tissues. Samples were homogenized using an Ultra Turrax homogenizer (John Morris, Sydney, Australia) and then centrifugated (12,000× *g*, 12 min, 4 °C) to extract the protein fraction. The supernatant of each sample was collected and used for analysis. The protein content of each sample was determined by Lowry analysis, as described elsewhere [47]. Spectrophotometric determinations were performed (wavelength 750 nm) after colorimetric reaction of the extracted proteins and the reactive mix (NaOH, 0.5 M; Folin-Ciocalteu reactive, CuSO_4_ × 5 H_2_O, Rochelle salt, and Na_2_CO_3_).

Superoxide dismutase (SOD) was determined on the S9 fraction mixed with Tris-EDTA buffer (pH 8.2) and pyrogallol [48]. This method is an indirect determination, as it is based on the ability of the SOD enzyme, if present in tissues, to inhibit pyrogallol autoxidation quantified spectrophotometrically at 420 nm and expressed as U mg^−1^ of protein.

Lipid peroxidation was tested using 10% of the extracted S9 protein fraction; this colorimetric reaction was conducted using phosphoric acid 1% (*v*/*v*) and thiobarbituric acid 0.6% (*w*/*v*), as described elsewhere [49]. The mix was heated and centrifuged in 1-buthanol, and the malondialdehyde (MDA) was quantified spectrophotometrically at wavelengths between 535 and 520 nm (1 nm minimum resolution); the results were expressed as U mg^−1^ of protein.

Glutathione peroxidase levels (GPx) were determined on 0.5 mg mL^−1^ of the extracted S9 protein fraction using a reaction mix of GSH (10 mM, reduced glutathione), 2.4 U mL^−1^ of GSSG reductase, and 1.5 mM NADPH, as described elsewhere [50]. The reaction mix was heated to 37 °C in hydrogen peroxide; GPx activity was quantified spectrophotometrically at 340 nm and read for 2 min. The results were expressed as nmol/(mg × min).

Glutathione-S-transferase (GST) analysis was performed by reacting the protein fraction and the mix of GSH (10 mM, reduced glutathione) and CDNB (60 mM, 1-chloro-2,4-dinitrobenzene) read at 340 nm for 5 min [51]; the results were expressed as nmol/(mg × min).

To integrate the results of the biomarkers, the integrated biological response version 2 index (IBRv2) was calculated according to Sanchez et al. [52]. Lower or higher index core values can be translated into the impact of treatment on organisms: higher index core values indicate poorer health status (stressed organisms).

#### 2.2.8. Chemical Determination of Phthalates by High-Performance Liquid Chromatography (HPLC-UV-Vis)

Water samples from each aquarium collected at the end of the exposure period were analyzed to determine phthalate release from microplastics. The extraction method and instrumental determination were optimized to our HPLC instrument, as reported elsewhere [53]. Briefly, analyses were performed on a Vanquish Core HPLC system (Thermo Fisher Scientific), equipped with a diode array detector (DAD, mod. CG) and a C18 column (Supelco^®^ Sigma-Aldrich) interfaced with Chromeleon^®^ software (Thermo Fischer Scientific) for optimizing runs according to pressure to determine DMP (dimethyl phthalate), DBP (dibutyl phthalate), and DNOP (di-n-octyl phthalate); the LOQ was 0.06 µg mL^−1^.

### 2.3. Statistical Analysis

Normality and homoscedasticity were assessed through the Shapiro-Wilk and Levene tests, respectively. The Mann-Whitney U test was used to detect differences in microplastics size of the unconditioned PP vs the conditioned PP. Mortality and biochemical responses were analyzed using the non-parametric Kruskal-Wallis test since the null hypothesis for normal distribution could not be rejected. Differences were considered significant at a *p*-value < 0.05. The Conover-Iman posthoc test was used to reveal differences within treatments and between treatments and controls. Statistical analysis was performed using RStudio version 3.5.3. Excel software (Microsoft, Redmond, WA, USA) was used for calculating the IBRv2 index and creating the star plots.

## 3. Results

**Figure 2:**
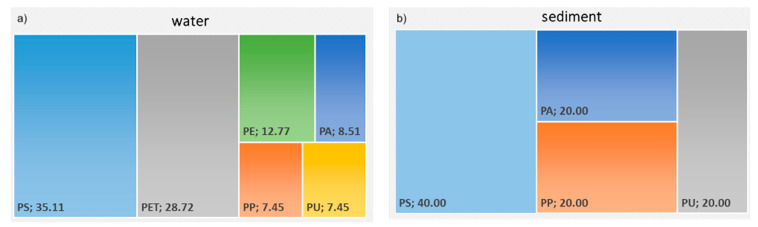
Chemical composition of microplastics (in percentage of abundance) detected in water (**a**) and sediment (**b**) samples (*n* = 3) from the Vipacco River. PA denotes polyamide; PP polypropylene; PET polyethylene terephthalate, PU polyurethane; PS polystyrene; PE polyethylene.

Both water and sediment from Vipacco river were contaminated by microplastics (3.73 ± 2.11 microplastics m^−3^ per min and 3.33 ± 4.16 microplastics dm^−3^, respectively). The chemical composition of the microplastics varied and included up to six different polymers (polystyrene-PS, polyethylene terephthalate-PET, polyurethane-PU, polyamide-PA, polypropylene-PP and polyethylene-PE). Figure 2 presents the relative abundance (%) of each polymer type. The most abundant polymer in the water samples was PS (35.11%), followed by PET (28.72%), PE (12.77%), PA (8.51%), PP and PU (each 7.45%). The mean size of microplastics was 463.2 ± 15.7 µm. Fibers (46%), spherules (39%) and fragments (15%) were the most dominant microplastics shape. In the sediment samples, the most abundant polymer was PS (40%), followed by PP, PA, and PU (each 20%). The mean size of microplastics was 141 ± 264 µm. The most frequent microplastics shape were fibers (42%) followed by spherules (50%) and fragments (8%). Blue, white and brown were the most abundant color in both water and sediment samples.

### 3.1. FT-IR Characterization of Conditioned Microplastics (PP-River; PP-sf vs. PP)

The microplastics for in vivo exposure were preconditioned in riverine water (PP-river) and in the surfactant Triton X-100 solution (PP-sf), then characterized by µFT-IR to determine the size and spectral changes. Dimensional analysis on 20 particles per treatment showed homogeneous particle size without statistically significant differences between the unconditioned PP and the PP conditioned in Vipacco riverine water and Triton X-100 solution (Mann-Whitney U test, *p* > 0.05) (Table 1).

**Figure 3:**
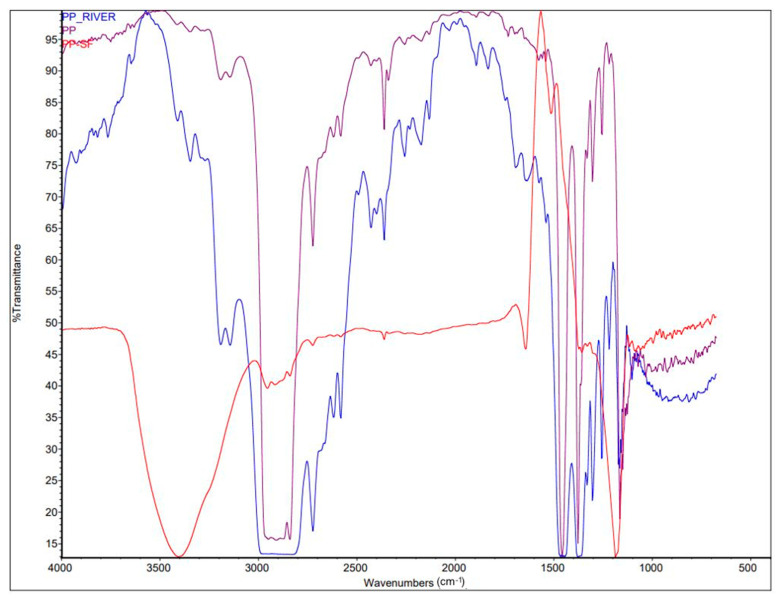
µFT-IR mean spectra (*n* = 10) acquired in transmission mode of unconditioned microplastics (PP, violet) vs. microplastics conditioned in riverine water (PP-river, blue) vs. microplastics conditioned in surfactant solution (PP-sf, red).

Comparison of mean spectra (*n* = 10) between conditioned microplastics (PP-river; PP-sf) and unconditioned microplastics (PP) (Figure 3) showed significant differences for the PP-sf; spectral matching was 8.54%, indicating release of chemicals from the microplastics into the water and absorption of surfactants onto the microplastics surface, whereas spectral matching for the PP-river was 78.59%.

### 3.2. In Vivo Experiment

The toxicity of conditioned and unconditioned microplastics was determined by measuring the percentage of mortality and the biochemical response of oxidative stress biomarkers (SOD, GPx, GST and MDA). The physiochemical water parameters were maintained constant throughout the experiment: Control−: pH (8.23 ± 0.03 pH), temperature (12.52 ± 0.48 °C), oxygen (94.27% ± 0.58); Control+: pH(8.12 ± 0.21) water temperature (12.89 ± 0.53 °C) and oxygen saturation (87.10 ± 2.96%); PP: pH (8.05 ± 0.05 pH), temperature (12.12 ± 0.48 °C), oxygen (91.22% ± 0.57); PP-river: pH (8.15 ± 0.06 pH), temperature (12.34 ± 0.48 °C), oxygen (92.21% ± 0.53); PP-sf: pH (8.08 ± 0.10 pH), temperature (12.45 ± 0.12 °C), oxygen (93.21% ± 0.46).

#### 3.2.1. Lethal Effects (Mortality)

**Figure 4:**
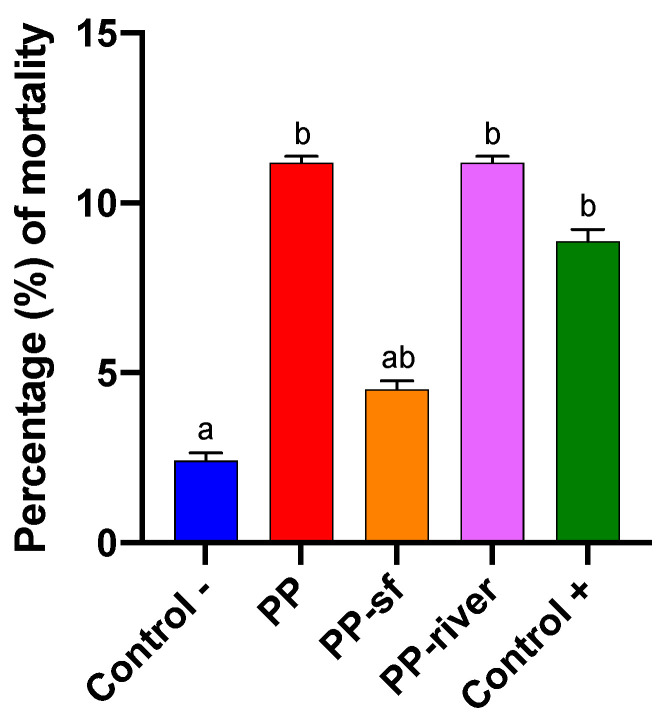
Mortality of *Hydropsyche pellucidula* for each treatment group (Control− denotes positive control; PP polypropylene unconditioned; PP-sf polypropylene preconditioned in Triton X-100 solution; PP-river polypropylene preconditioned in riverine water; Control+ negative control) expressed as percentage (%). Lowercase letters (a, b) denote differences revealed by Conover-Iman post-hoc test.

Figure 4 presents the mortality rate for each treatment group. The percentage (%) of deaths in the Control− group was far lower (2.22%) than in the other treatment groups. The mortality rate for the PP and the PP-river group was 11.11%, followed by the Control+ (8.89%) and the PP-sf group (4.44%). The non-parametric Kruskal-Wallis test revealed significant differences between the Control− group and the treated groups (*p* < 0.001). Conover-Iman multiple comparisons test revealed significant differences between Control− and PP (*p* = 0.03), Control− and PP-river (*p* = 0.03), Control− and Control+ (*p* = 0.04).

**Figure 5:**
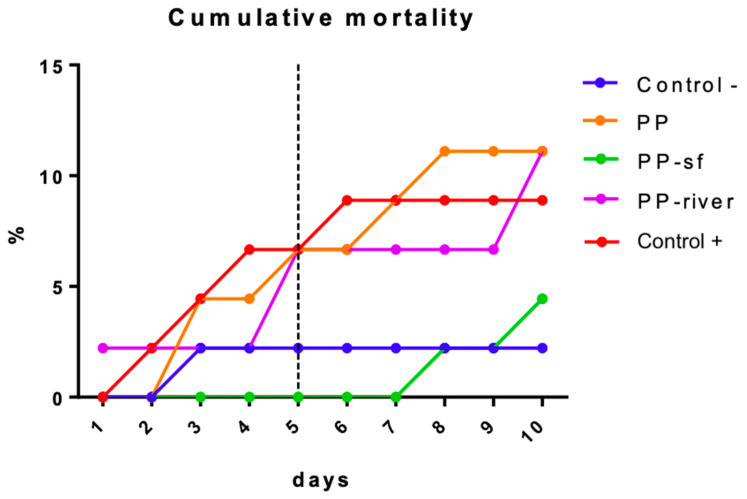
Cumulative mortality (in percentage; average values of three replicates) of *Hydropsyche pellucidula* during exposure in the four treatment groups (Control−, PP, PP-sf, PP-river, Control+). The dashed line indicates the midpoint of the experiment. Control− denotes negative control; PP polypropylene unconditioned; PP-sf denotes polypropylene preconditioned in Triton X-100 solution; PP-river polypropylene preconditioned in riverine water; Control+ positive control.

Figure 5 shows the daily mortality rate for each experimental group: the rate was similar (6.67%) for the Control+, the PP, and the PP-river group in 5 days of exposure; the rate for the Control− group remained constant from the third day onwards, whereas the rate for the PP-sf group started at 1 week of exposure and surpassed that of the C− group by the end of the experiment.

#### 3.2.2. Sublethal Effects

**Figure 6:**
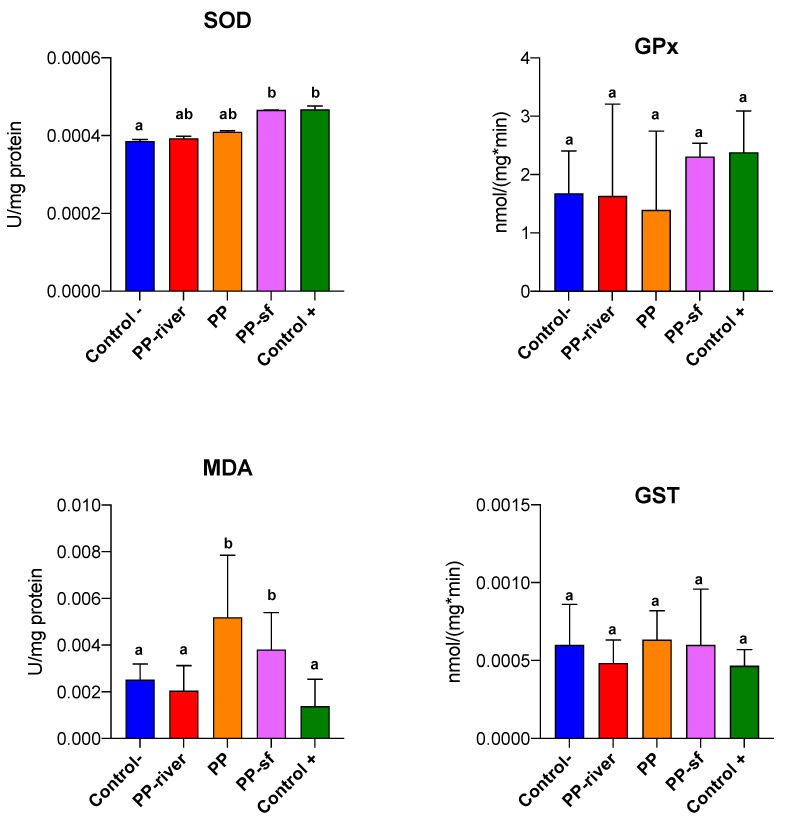
Oxidative stress biomarkers in *Hydropsyche pellucidula* larvae exposed to treatments for 10 days. SOD (superoxide dismutase; U/mg protein); GPx (glutathione peroxidase; nmol/min mg protein); MDA (malondialdehyde; U/mg protein), GST (glutathione S-transferase; nmol/min mg protein). Mean ± standard deviation, *n* = 6. Control− denotes negative control; PP polypropylene unconditioned; PP-sf denotes polypropylene preconditioned in Triton X-100 solution; PP-river polypropylene pre-conditioned in riverine water; Control+ positive control. Lowercase letters (a, b) denote differences revealed by Conover–Iman posthoc test.

Analysis of the C− and the treatment groups by means of the Kruskal–Wallis test on live larvae at the end of the exposure period revealed no statistical difference in biochemical response for GPx and GST (*p* = 0.13 and 0.28, respectively) (Figure 6), whereas there were statistically significant differences for MDA (*p* = 0.002) and SOD (*p* = 0.009). The Conover-Iman posthoc test showed differences between the Control− and Control+ (*p* = 0.009) and between Control- and PP-sf (*p* = 0.032) for SOD. Also, it highlighted differences between C− and PP (*p* = 0.019), between C− and PP-sf (*p* = 0.019), between PP-river and PP (*p* = 0.019), between PP-river and PP-sf (*p* = 0.019), between C+ and PP (*p* = 0.001), between C+ and PP-sf (*p* = 0.018) for MDA.

**Figure 7:**
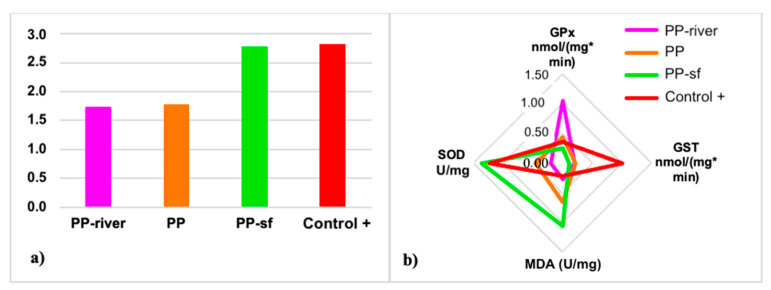
(**a**) IBRv2 for each treatment. (**b**) Star plot based on the reference deviation of each biomarker: SOD, superoxide dismutase; GPx, glutathione peroxidase; MDA, malondialdehyde; GST, glutathione S-transferase. Control− denotes negative control; PP polypropylene unconditioned; PP-sf denotes polypropylene preconditioned in Triton X-100 solution; PP-river polypropylene pre-conditioned in riverine water; Control+ positive control.

To facilitate the interpretation of the biochemical response and gain a better picture of the overall health status of larvae after exposure to the treatments, the IBRv2 index was calculated. The *Hydropsyche* larvae showed a global deterioration in health status compared to the Control− group: IBRv2 index 2.81, 2.78, 1.77, and 1.73 for the Control+, the PP-sf, the PP, and the PP-river group, respectively (Figure 7a,b). Finally, all treatments except PP-river seemed to modulate the activation of SOD and MDA (Figure 7b).

#### 3.2.3. Microplastics in Biota

No microplastics were detected in the *H. pellucidula* after exposure to any of the treatments.

#### 3.2.4. Phthalate Release from Microplastics

Water samples collected from each aquarium showed phthalate values lower than the LOQ.

## 4. Discussion

To understand the effects of microplastics on freshwater ecosystems, real environmental samples need to be collected and analyzed for microplastics abundance, shape, and composition. In the first part of this study, the level of microplastics contamination was assessed in different environmental matrices (sediment and water) from the Vipacco River. The findings revealed that riverine water was the most polluted compartment (3.73 microplastics/m^3^/min). The water samples were also characterized by a certain variability in the chemical composition of microplastics, including not only buoyant polymers (PS, PA, PP, PE) but also high-density plastics (PET and PU). These outcomes show that density is just one of the many parameters (in addition to particle size, human population density, economic and urban development, waste management, hydrological conditions) that can influence the fate and vertical transport of microplastics [54].

In Vipacco River, fibers accounted for the main type of microplastics detected in the water and the sediment samples (46% and 42%, respectively). This finding is shared by previous studies (i.e., [55,56]) that reported that fibers were the most abundant microplastics in abiotic samples. The fibers settle on the riverbed when the flow velocity is too slow to keep them suspended [57], making them available for ingestion by aquatic organisms [58]. According to available data [42], PP and PE are the main microplastic type recorded in water and sediments. However, the main microplastic type found in Vipacco river in both water and sediment samples was PS, which is widely used in construction materials, packaging foam, food containers, clerical supplies, medical equipment, fishing gear and many other applications.

Literature results about microplastic concentration show high variability of the protocols used and therefore, in the units of measurement in which the results are expressed (in both water and sediment studies), making it difficult to compare with our findings [42]. A recent study focused on the microplastic occurrence in an Italian watercourse (Ofanto river, southeast Italy) [59] found a concentration (expressed as a mean value of six replicates ± standard deviation) that ranged from 0.9 ± 0.4 items/m^3^ to 13 ± 5 items /m^3^, in line with our results. On the other hand, a comparison of microplastic concentration in sediment from Vipacco river with other studies is not possible, since the only available study on sediment from lotic freshwater reported values expressed as items/kg [60].

*Hydropsyche pellucidula* is considered both a collector-gatherer and filterer organism [32]. Thus, it is important to know the characteristics of microplastics in both water and sediment samples. On this path, data from the environmental samples were then used for laboratory exposure of microplastics preconditioned in riverine water (PP-river) and in a surfactant solution (PP-sf). According to FT-IR analysis, the PP-sf group underwent major spectral changes. These outcomes presage a difference in toxicity between PP-river and PP-sf, as demonstrated by the toxicological test on *H. pellucidula* larvae. On this path, Renzi et al. [61] showed how the addition of the surfactant Triton X-100 to microplastic particles produced significant effects on both mortality and immobilization of *Daphnia magna*, in line with results here reported.

*Hydropsyche* larvae may provide a good model organism for freshwater macroinvertebrates since they are easy to collect in the field, are highly abundant in their habitat, are easy to house (trial test demonstrated their tolerance to captivity, with no signs of visible stress and extremely low mortality), and their responsiveness to chemical stress simulated in this study. Statistical tests on mortality disclose differences among treatments and interesting biological findings were noted. As concerns, the lethal effects, unconditioned (PP) and PP-river (the most hazardous treatments) were associated with a five-fold increase in mortality compared to the negative control. The equal mortality percentage after such treatments suggests that submersion for 15 days in river water was insufficient to influence the sorption dynamic between natural water and PP.

On this path, Klein et al. [62] in a study focused on the exposure of the freshwater oligochaete *Lumbriculus variegatus* to biodegradable microplastics showed how solvent-treated microplastics are less toxic than untreated microplastics and how exposure to methanolic extracted chemicals from microplastics induces strong toxicity, confirming our results. Although the phthalate concentrations in water samples were below the LOQ, preconditioning of PP in Triton X-100 probably caused the release of weakly bound contaminants, reducing the lethal effect to values similar to the negative control. It is likely that the Triton X-100 surfactant washed out lipophilic substances from the PP [63].

Finally, the third most hazardous treatment was exposure to Triton X-100 solution, which increased the mortality rate fourfold. Surfactants are widespread in aquatic environments [64] and can show direct toxicity on aquatic species [65]. Indeed, the toxicity of surfactants is primarily a function of their intrinsic property to adsorb and penetrate the cell membrane of living organisms [66]. Non-ionic surfactants may cause serious damage to freshwater organisms, including death in *Daphnia magna* (Crustacea, Cladocera; LC_50_ 201 µg L^−1^, 48 h) [67] and *Physa acuta* (Gastropoda, Physidae; LC_50_ 4.69–5.33 mg/L, 24 h) [68]. Despite the extremely low concentration of Triton X-100 used in the present study (10 µg L^−1^), exposure for 10 days was found to be potentially hazardous for the genus *Hydropsyche*.

A different evolution over time between treatments was noted. The lethal effects of exposure to Control+, PP, and PP-river were observed in the first half of the exposure period, resulting in 75% and 60% of total mortality at day 5, respectively. Differently, the mortality rate in the Control− remained constant from the third day onwards, and exposure to PP-sf started to induce lethal effects at one week, surpassing the C− by the end of the experiment. Further studies on a wider range of Triton X-100 concentrations are necessary to better characterize its ecotoxicity.

While the surfactant was probably able to remove compounds from the microplastics surface and reduce the lethal PP toxicity, it exposed the larvae to PP that was impoverished but perhaps even more prone to the release of superoxide anion (O_2_^−^) and trigger oxidative stress [69].

One of the recognized mechanisms of toxicity of surfactants is oxidative stress leading to a loss of fluidity and increased ion permeability of the biological membrane. Octyl phenol ethoxylate (Triton X-100) is a nonionic surfactant with a hydrophobic chain and hydrocarbon group. Indeed, hydrocarbon metabolisms of surfactants by aquatic organisms can boost cellular ROS concentration leading to the disruption of membrane integrity. Moreover, chemical and physicochemical parameters of surfactants can alter the biological activity of proteins and peptides modifying the folding of the polypeptide chain and the surface charge of the macromolecule [70]. Even size and dose of microplastics and/or interaction with other contaminants increase lipid peroxidation inducing oxidative damage and altering oxidative stress biomarkers [71].

Although the mechanisms of oxidative stress induction on aquatic organisms are still debated, the first antioxidant defense line (SOD, CAT and GSH) can act as a good biomarker for evaluating early oxidative damage induced by microplastics [71].

In the present study, PP-sf altered the levels of several of the tested biomarkers. SOD and MDA were the most prominent and insightful oxidative stress biomarkers with higher levels recorded for the PP-sf treated larvae at the end of the exposure period. Unlike PP-sf, the other treatments conditions did not markedly affect SOD activity and hence the production of superoxide anions may not act as a primary factor of oxidative impairment.

Evidence of an increase in lipid peroxidation was observed in the PP-sf group. While for SOD, oxidative stress induced by microplastics previously treated with surfactant can be linked to the residual surfactant (similar values), the same cannot be said for MDA: the MDA values for PP-sf were more than 16 times greater than treatment with the surfactant alone (C+).

Elevated values of lipid hydroperoxide (LPO) following the failure of antioxidant defenses can be recorded in organisms exposed to environmental contaminants [71]. Malondialdehyde (MDA) is a by-product of LPO widely used as an oxidative stress biomarker. To compute the overall stress of all treatments on *H. pellucidula* larvae the IBRv2 index was applied and SOD and LPO were the most sensitive biomarkers. IBRv2 can successfully discriminate toxicity among various contaminants thus providing a broader integrated view of biomarkers response [52]. It is a reliable tool to better categorize the severity of stressors on organisms and to summarize biomarker responses. The integrated results of IBRv2 suggest a worsening of the health status of larvae exposed to Control+ and PP-sf groups, compared to the negative control. Indeed, activation of SOD activity indicates antioxidant shielding against oxidative pressure; however, it was not entirely efficient to counteract ROS concentration and lipid peroxidation in PP-sf treatment. Treatments that included a surfactant seemed to create more stressful conditions than surfactant-free treatments.

Finally, a slightly but non-significant higher level of GST was observed in *H. pellucidula* larvae exposed to unconditioned PP. This finding is in line with results reported by Lei et al. [72] which found an increase in GST 4 enzyme gene expression in the intestine of the benthic aquatic nematode *Caenorhabditis elegans* exposed to PP.

The absence of microplastics in the biota of the larvae suggests that ecotoxicological effects might be related less to plastics ingestion and more to the chemical risk or to the abrasion effect on the external surfaces of the larvae. In a recent study, Bertoli et al. [73] showed how freshwater macroinvertebrates can ingest a wide range of microplastic sizes (mean ± standard deviation: 141 ± 264 μm). On this path, microplastics can be ingested and excreted rapidly, even in less than an hour [74], passing through the digestive tract and gut lumen, being excreted through feces [58]. The ingestion of microplastics by macroinvertebrates is not fully explained simply by an abundance of microplastics but also depends on the characteristics of the microplastics (i.e., size, density, shape, polymer), as well as biological factors and life-history traits [58,75]. Moreover, the ingestion is also affected by the environment [76]. Indeed, the aging of microplastics in the environment promotes ingestion, with negative effects on organisms being dose-dependent [77]. Based on such an assumption, significant negative effects on organisms occur only when the microplastics dose with which organisms are challenged exceeds organism-specific toxicological thresholds [76]. Furthermore, a recent study highlighted how caddisflies and mayflies seem to use mainly microplastics over natural construction materials or substrates [78], threatening caddisflies by destabilizing their cases [79].

## 5. Conclusions

As the world’s human population expands, the rates of plastics production increase, therefore, plastic and microplastic represent a growing threat to the health of freshwater ecosystems which are recognized as origins and transport pathways of plastics to the oceans. In this context, it was deemed of interest to investigate microplastic effects on freshwater macroinvertebrates, since they occupy a central role in the freshwater environments. The findings of our study may enhance knowledge on the toxicity of commercial PP and the conditioning phase of plastics on *H. pellucidula* larvae. The absence of PP microplastics in the larvae suggests that ecotoxicological effects might be related less to plastic ingestion and more to the chemical risk or to the abrasion effect on the external surfaces of the larvae. On this path, the mortality rate was significantly higher in larvae exposed to PP and PP-river, indicating negative effects of microplastics on *H. pellucidula* larvae. Moreover, analysis of oxidative stress biomarker levels showed a greater response of SOD and MDA in larvae treated with PP-sf. Further studies are needed to better assess the effects of other microplastics concentrations, chemical types, and sizes on the caddisfly *Hydropsyche*.

## Figures and Tables

**Table 1:** μFT-IR dimensional analysis (mean, standard deviation, minimum and maximum). Comparison of particles (*n* = 20 per group) (Mann-Whitney U test) after pre-treatment in three different solutions. PP denotes polypropylene unconditioned; PP-river polypropylene preconditioned in riverine water; PP-sf polypropylene preconditioned in Triton X-100 solution.

Microplastic	Mean (µm)	Standard Deviation	Min (µm)	Max (µm)	*p*-Value
PP	377.7	168.0	179.7	497.8	-
PP-river	381.8	100.9	177.0	487.1	0.89
PP-sf	368.0	116.2	185.7	502.0	0.80

## References

[1] Bergmann M., Gutow L., Klages M. (2015). Marine Anthropogenic Litter.

[2] Hartmann N.B., Hüffer T., Thompson R.C., Hassellov M., Verschoor A., Daugaard A.E., Rist S., Karlsson T., Brennholt N., Cole M. (2019). Are We Speaking the Same Language? Recommendations for a Definition and Categorization Framework for Plastic Debris. Environ. Sci. Technol..

[3] Ng E.-L., Lwanga E.H., Eldridge S.M., Johnston P., Hu H.-W., Geissen V., Chen D. (2018). An overview of microplastic and nanoplastic pollution in agroecosystems. Sci. Total Environ..

[4] Imhof H.K., Ivleva N.P., Schmid J., Niessner R., Laforsch C. (2013). Contamination of beach sediments of a subalpine lake with microplastic particles. Curr. Biol..

[5] Sighicelli M., Pietrelli L., Lecce F., Iannilli V., Falconieri M., Coscia L., Di Vito S., Nuglio S., Zampetti G. (2018). Microplastic pollution in the surface waters of Italian Subalpine Lakes. Environ. Pollut..

[6] Pastorino P., Pizzul E., Bertoli M., Anselmi S., Kušće M., Menconi V., Prearo M., Renzi M. (2021). First insights into plastic and microplastic occurrence in biotic and abiotic compartments, and snow from a high-mountain lake (Carnic Alps). Chemosphere.

[7] Rodrigues M.O., Abrantes N., Gonçalves F.J.M., Nogueira H., Marques J.C., Gonçalves A.M.M. (2018). Spatial and temporal distribution of microplastics in water and sediments of a freshwater system (Antuã River, Portugal). Sci. Total Environ..

[8] Wang C., Xing R., Sun M., Ling W., Shi W., Cui S., An L. (2020). Microplastics profile in a typical urban river in Beijing. Sci. Total Environ..

[9] Morritt D., Stefanoudis P.V., Pearce D., Crimmen O.A., Clark P.F. (2014). Plastic in the Thames: A river runs through it. Mar. Pollut. Bull..

[10] Mintenig S.M., Löder M.G.J., Primpke S., Gerdts G. (2019). Low numbers of microplastics detected in drinking water from ground water sources. Sci. Total Environ..

[11] Talvitie J., Mikola A., Setälä O., Heinonen M., Koistinen A. (2017). How well is microlitter purified from wastewater?—A detailed study on the stepwise removal of microlitter in a tertiary level wastewater treatment plant. Water Res..

[12] Blettler M.C., Abrial E., Khan F.R., Sivri N., Espinola L.A. (2018). Freshwater plastic pollution: Recognizing research biases and identifying knowledge gaps. Water Res..

[13] Koelmans A.A., Nor N.H.M., Hermsen E., Kooi M., Mintenig S.M., De France J. (2019). Microplastics in freshwaters and drinking water: Critical review and assessment of data quality. Water Res..

[14] Horton A.A., Walton A., Spurgeon D.J., Lahive E., Svendsen C. (2017). Microplastics in freshwater and terrestrial environments: Evaluating the current understanding to identify the knowledge gaps and future research priorities. Sci. Total Environ..

[15] Blarer P., Burkhardt-Holm P. (2016). Microplastics affect assimilation efficiency in the freshwater amphipod Gammarus fossarum. Environ. Sci. Pollut. Res..

[16] Au S.Y., Bruce T.F., Bridges W.C., Klaine S.J. (2015). Responses of *Hyalella azteca* to acute and chronic microplastic exposures. Environ. Toxicol. Chem..

[17] Rehse S., Kloas W., Zarfl C. (2016). Short-term exposure with high concentrations of pristine microplastic particles leads to immobilisation of *Daphnia magna*. Chemosphere.

[18] Karami A., Groman D.B., Wilson S.P., Ismail P., Neela V.K. (2017). Biomarker responses in zebrafish (*Danio rerio*) larvae exposed to pristine low-density polyethylene fragments. Environ. Pollut..

[19] Qiao R., Sheng C., Lu Y., Zhang Y., Ren H., Lemos B. (2019). Microplastics induce intestinal inflammation, oxidative stress, and disorders of metabolome and microbiome in zebrafish. Sci. Total Environ..

[20] Bhagat J., Zang L., Nishimura N., Shimada Y. (2020). Zebrafish: An emerging model to study microplastic and nanoplastic toxicity. Sci. Total Environ..

[21] Bhagat J., Nishimura N., Shimada Y. (2021). Toxicological interactions of microplastics/nanoplastics and environmental contaminants: Current knowledge and future perspectives. J. Hazard. Mater..

[22] Magara G., Khan F.R., Pinti M., Syberg K., Inzirillo A., Elia A.C. (2019). Effects of combined exposures of fluoranthene and polyethylene or polyhydroxybutyrate microplastics on oxidative stress biomarkers in the blue mussel (*Mytilus edulis*). J. Toxicol. Environ. Health Part A.

[23] Magara G., Elia A.C., Syberg K., Khan F.R. (2018). Single contaminant and combined exposures of polyethylene microplastics and fluoranthene: Accumulation and oxidative stress response in the blue mussel, *Mytilus edulis*. J. Toxicol. Environ. Health Part A.

[24] Rochman C.M., Bergmann M., Gustow L., Klages M. (2015). The complex mixture, fate and toxicity of chemicals associated with plastic debris in the marine environment. Marine Anthropogenic Litter.

[25] Bellasi A., Binda G., Pozzi A., Galafassi S., Volta P., Bettinetti R. (2020). Microplastic Contamination in Freshwater Environments: A Review, Focusing on Interactions with Sediments and Benthic Organisms. Environments.

[26] Oehlmann J., Schulte-Oehlmann U., Kloas W., Jagnytsch O., Lutz I., Kusk K.O., Wollenberger L., Santos E.M., Paull G.C., Van Look K.J.W. (2009). A critical analysis of the biological impacts of plasticizers on wildlife. Philos. Trans. R. Soc. B Biol. Sci..

[27] Sakai S., Urano S., Takatsuki H. (2000). Leaching behavior of PCBs and PCDDs/DFs from some waste materials. Waste Manag..

[28] Cirelli A.F., Ojeda C., Castro M.J., Salgot M., Lichtfouse E. (2009). Surfactants in sludge-amended agricultural soils: A review. Organic Farming, Pest Control and Remediation of Soil Pollutants.

[29] Pastorino P., Brizio P., Abete M.C., Bertoli M., Noser A.G.O., Piazza G., Prearo M., Elia A.C., Pizzul E., Squadrone S. (2020). Macrobenthic invertebrates as tracers of rare earth elements in freshwater watercourses. Sci. Total Environ..

[30] Haegerbaeumer A., Mueller M.-T., Fueser H., Traunspurger W. (2019). Impacts of micro-and nano-sized plastic particles on benthic invertebrates: A literature review and gap analysis. Front. Environ. Sci..

[31] Fueser H., Mueller M.-T., Traunspurger W. (2020). Ingestion of microplastics by meiobenthic communities in small-scale microcosm experiments. Sci. Total Environ..

[32] Merritt R.W., Cummins K.W. (1996). An Introduction to the Aquatic Insects of North America.

[33] Geraci C.J., Zhou X., Morse J.C., Kjer K.M. (2010). Defining the genus *Hydropsyche* (Trichoptera: Hydropsychidae) based on DNA and morphological evidence. J. N. Am. Benthol. Soc..

[34] Awrahman Z.A., Rainbow P.S., Smith B.D., Khan F.R., Fialkowski W. (2016). Caddisflies *Hydropsyche* spp. as biomonitors of trace metal bioavailability thresholds causing disturbance in freshwater stream benthic communities. Environ. Pollut..

[35] Macedo-Sousa J.A., Gerhardt A., Brett C.M., Nogueira A.J., Soares A.M. (2008). Behavioural responses of indigenous benthic invertebrates (*Echinogammarus meridionalis*, *Hydropsyche pellucidula* and *Choroterpes picteti*) to a pulse of acid mine drainage: A laboratorial study. Environ. Pollut..

[36] Wendt-Rasch L., Vought L.B.-M., Woin P. (1998). Effects of fenvalerate on the net-spinning behaviour of *Hydropsych siltalai* (Döhler) (Trichoptera: Hydropsychidae). Hydrobiologia.

[37] Tessier L., Boisvert J.L., Vought L.B.M., Lacoursie J.O. (2000). Effects of 2, 4-dichlorophenol on the net-spinning behavior of *Hydropsyche slossonae* larvae (Trichoptera; Hydropsychidae), an early warning signal of chronic toxicity. Ecotoxicol. Environ. Saf..

[38] Mosetti F. (1983). Sintesi sull’idrologia del Friuli-Venezia Giulia. Quad. dell’Ente Tutela Pesca.

[39] Scherer C., Weber A., Stock F., Vurusic S., Egerci H., Kochleus C., Arendt N., Foeldi C., Dierkes G., Wagner M. (2020). Comparative assessment of microplastics in water and sediment of a large European river. Sci. Total Environ..

[40] Enders K., Lenz R., do Sul J.A.I., Tagg A.S., Labrenz M. (2020). When every particle matters: A QuEChERS approach to extract microplastics from environmental samples. MethodsX.

[41] Buffagni A., Erba S., Genoni P., Lucchini D., Orlandi C. (2014). Protocollo di campionamento e analisi dei macroinvertebrati bentonici dei corsi d’acqua guadabili. ISPRA Manuali e Linee Guida.

[42] Cera A., Cesarini G., Scalici M. (2020). Microplastics in Freshwater: What Is the News from the World?. Diversity.

[43] Rummel C.D., Jahnke A., Gorokhova E., Kühnel D., Schmitt-Jansen M. (2017). Impacts of biofilm formation on the fate and potential effects of microplastic in the aquatic environment. Environ. Sci. Technol. Lett..

[44] Xia Y., Zhou J.J., Gong Y.Y., Li Z.J., Zeng E.Y. (2020). Strong influence of surfactants on virgin hydrophobic microplastics adsorbing ionic organic pollutants. Environ. Pollut..

[45] Renzi M., Bertoli M., Pastorino P., Pizzul E. Microplastiche nel fiume Vipacco.

[46] De Marchi L., Neto V., Pretti C., Figueira E., Brambilla L., Rodriguez-Douton M.J., Rossella F., Tommasini M., Furtado C., Soaresa A.M.V.M. (2017). Physiological and biochemical impacts of graphene oxide in polychaetes: The case of *Diopatra neapolitana*. Comp. Biochem. Phys. Part C Toxicol. Pharmacol..

[47] Dubois M., Gilles K.A., Hamilton J.K., Rebers P.A., Smith F. (1956). Colorimetric method for determination of sugars and related substances. Anal. Chem..

[48] Gao R., Yuan Z., Zhao Z., Gao X. (1998). Mechanism of pyrogallol autoxidation and determination of superoxide dismutase enzyme activity. Bioelectrochem. Bioenerg..

[49] Uchiyama M., Mihara M. (1978). Determination of malonaldehyde precursor in tissues by thiobarbituric acid test. Anal. Biochem..

[50] Badary O.A., Abdel-Maksoud S., Ahmed W.A., Owieda G.H. (2005). Naringenin attenuates cisplatin nephrotoxicity in rats. Life Sci..

[51] Habig W.H., Pabst M.J., Jakoby W.B. (1974). Glutathione S-transferases: The first enzymatic step in mercapturic acid formation. J. Biol. Chem..

[52] Sanchez W., Burgeot T., Porcher J.M. (2013). A novel “Integrated Biomarker Response” calculation based on reference deviation concept. Environ. Sci. Pollut. Res..

[53] Jing C., Qun X., Rohrer J. (2006). Determination of phthalates in drinking water by UHPLC with UV detection. Matrix.

[54] Wu P., Huang J., Zheng Y., Yang Y., Zhang Y., He F., Chen H., Quan G., Yan J., Li T. (2019). Environmental occurrences, fate, and impacts of microplastics. Ecotoxicol. Environ. Saf..

[55] Nel H.A., Dalu T., Wasserman R.J. (2018). Sinks and sources: Assessing microplastic abundance in river sediment and deposit feeders in an austral temperate urban river system. Sci. Total Environ..

[56] Akindele E.O., Ehlers S.M., Koop J.H.E. (2020). Freshwater insects of different feeding guilds ingest microplastics in two Gulf of Guinea tributaries in Nigeria. Environ. Sci. Pollut. Res..

[57] Voshell J.R., Wright A.B. (2002). A Guide to Common Freshwater Invertebrates of North America.

[58] Windsor F.M., Tilley R.M., Tyler C.R., Ormerod S.J. (2019). Microplastic ingestion by riverine macroinvertebrates. Sci. Total Environ..

[59] Campanale C., Stock F., Massarelli C., Kochleus C., Bagnuolo G., Reifferscheid G., Uricchio V.F. (2019). Microplastics and their possible sources: The example of Ofanto river in Southeast Italy. Environ. Pollut..

[60] Guerranti C., Cannas S., Scopetani C., Fastelli P., Cincinelli A., Renzi M. (2017). Plastic litter in aquatic environments of Maremma Regional Park (Tyrrhenian Sea, Italy): Contribution by the Ombrone river and levels in marine sediments. Mar. Pollut. Bull..

[61] Renzi M., Grazioli E., Blašković A. (2019). Effects of Different Microplastic Types and Surfactant-Microplastic Mixtures Under Fasting and Feeding Conditions: Case Study on *Daphnia magna*. Bull. Environ. Contam. Toxicol..

[62] Klein K., Piana T., Lauschke T., Schweyen P., Dierkes G., Ternes T., Schulte-Oehlmann U., Oehlmann J. (2021). Chemicals associated with biodegradable microplastic drive the toxicity to the freshwater oligochaete *Lumbriculus variegatus*. Aquat. Toxicol..

[63] Casteloes K.S., Mendis G.P., Avins H.K., Howarter J.A., Whelton A.J. (2017). The interaction of surfactants with plastic and copper plumbing materials during decontamination. J. Hazard. Mater..

[64] Renzi M., Giovani A., Focardi S.E. (2012). Water pollution by surfactants: Fluctuations due to tourism exploitation in a lagoon ecosystem. J. Environ. Prot..

[65] Lechuga M., Fernández-Serrano M., Jurado E., Núñez-Olea J., Ríos F. (2016). Acute toxicity of anionic and non-ionic surfactants to aquatic organisms. Ecotoxicol. Environ. Saf..

[66] Mungray A.K., Kumar P. (2008). Occurrence of anionic surfactants in treated sewage: Risk assessment to aquatic environment. J. Hazard. Mater..

[67] Morrall D.D., Belanger S.E., Dunphy J.C. (2003). Acute and chronic aquatic toxicity structure–activity relationships for alcohol ethoxylates. Ecotoxicol. Environ. Saf..

[68] Liwarska-Bizukojc E., Miksch K., Malachowska-Jutsz A., Kalka J. (2005). Acute toxicity and genotoxicity of five selected anionic and nonionic surfactants. Chemosphere.

[69] De Sá L.C., Oliveira M., Ribeiro F., Rocha L.T., Futter M.N. (2018). Studies of the effects of microplastics on aquatic organisms: What do we know and where should we focus our efforts in the future?. Sci. Total Environ..

[70] Li M.-H. (2008). Effects of nonionic and ionic surfactants on survival, oxidative stress, and cholinesterase activity of planarian. Chemosphere.

[71] Prokić M.D., Radovanovic T.B., Gavric J.P., Faggio C. (2019). Ecotoxicological effects of microplastics: Examination of biomarkers, current state and future perspectives. TrAC Trends Anal. Chem..

[72] Lei L., Wu S., Lu S., Liu M., Song Y., Fu Z., Shi H., Raley-Susman K.M., He D. (2018). Microplastic particles cause intestinal damage and other adverse effects in zebrafish *Danio rerio* and nematode *Caenorhabditis elegans*. Sci. Total Environ..

[73] Bertoli M., Pastorino P., Lesa D., Renzi M., Anselmi S., Prearo M., Pizzul E. (2021). Microplastics accumulation in functional feeding guilds and functional habit groups of freshwater macrobenthic invertebrates: Novel insights in a riverine ecosystem. Sci. Total Environ..

[74] Wang W., Gao H., Jin S., Li R., Na G. (2019). The ecotoxicological effects of microplastics on aquatic food web, from primary producer to human: A review. Ecotoxicol. Environ. Saf..

[75] Agathokleous E., Iavicoli I., Barceló D., Calabrese E.J. (2021). Ecological risks in a ‘plastic’world: A threat to biological diversity?. J. Hazard. Mater..

[76] Agathokleous E., Iavicoli I., Barceló D., Calabrese E.J. (2021). Micro/nanoplastics effects on organisms: A review focusing on ‘dose’. J. Hazard. Mater..

[77] Kalčíková G., Skalar T., Marolt G., Kokalj A.J. (2020). An environmental concentration of aged microplastics with adsorbed silver significantly affects aquatic organisms. Water Res..

[78] Gallitelli L., Cera A., Cesarini G., Pietrelli L., Scalici M. (2021). Preliminary indoor evidences of microplastic effects on freshwater benthic macroinvertebrates. Sci. Rep..

[79] Ehlers S.M., Al Najjar T., Taupp T., Koop J.H.E. (2020). PVC and PET microplastics in caddisfly (*Lepidostoma basale*) cases reduce case stability. Environ. Sci. Pollut. Res..

